# Exploring the Measurement Properties of the eHealth Literacy Scale (eHEALS) Among Baby Boomers: A Multinational Test of Measurement Invariance

**DOI:** 10.2196/jmir.5998

**Published:** 2017-02-27

**Authors:** Lynn Sudbury-Riley, Mary FitzPatrick, Peter J Schulz

**Affiliations:** ^1^ Management School University of Liverpool Liverpool United Kingdom; ^2^ Waikato Management School University of Waikato Hamilton New Zealand; ^3^ Institute of Communication and Health Faculty of Communication Sciences University of Lugano Lugano Switzerland

**Keywords:** health literacy, eHealth literacy, eHEALS, baby boomers, health information, measurement invariance

## Abstract

**Background:**

The eHealth Literacy Scale (eHEALS) is one of only a few available measurement scales to assess eHealth literacy. Perhaps due to the relative paucity of such measures and the rising importance of eHealth literacy, the eHEALS is increasingly a choice for inclusion in a range of studies across different groups, cultures, and nations. However, despite its growing popularity, questions have been raised over its theoretical foundations, and the factorial validity and multigroup measurement properties of the scale are yet to be investigated fully.

**Objective:**

The objective of our study was to examine the factorial validity and measurement invariance of the eHEALS among baby boomers (born between 1946 and 1964) in the United States, United Kingdom, and New Zealand who had used the Internet to search for health information in the last 6 months.

**Methods:**

Online questionnaires collected data from a random sample of baby boomers from the 3 countries of interest. The theoretical underpinning to eHEALS comprises social cognitive theory and self-efficacy theory. Close scrutiny of eHEALS with analysis of these theories suggests a 3-factor structure to be worth investigating, which has never before been explored. Structural equation modeling tested a 3-factor structure based on the theoretical underpinning to eHEALS and investigated multinational measurement invariance of the eHEALS.

**Results:**

We collected responses (N=996) to the questionnaires using random samples from the 3 countries. Results suggest that the eHEALS comprises a 3-factor structure with a measurement model that falls within all relevant fit indices (root mean square error of approximation, RMSEA=.041, comparative fit index, CFI=.986). Additionally, the scale demonstrates metric invariance (RMSEA=.040, CFI=.984, ΔCFI=.002) and even scalar invariance (RMSEA=.042, CFI=.978, ΔCFI=.008).

**Conclusions:**

To our knowledge, this is the first study to demonstrate multigroup factorial equivalence of the eHEALS, and did so based on data from 3 diverse nations and random samples drawn from an increasingly important cohort. The results give increased confidence to researchers using the scale in a range of eHealth assessment applications from primary care to health promotions.

## Introduction

The importance of health literacy for health status is well recognized. The American Medical Association, for example, found that health literacy has a stronger impact on health status than several sociodemographic variables [1] and is crucial in empowering patients to play a more active role in their own health care [2-4]. The Alliance for Health and the Future illustrates the significance of health literacy when describing it as an essential life skill for individuals, a public health imperative, an essential part of social capital, and a critical economic issue [5].

Health information is one of the most frequently sought topics on the Internet [6-8]. Consequently, in today’s networked environment, electronic health resources are becoming increasingly vital in terms of overall health literacy [9,10]. New technologies that open up a myriad of eHealth applications and communications channels are revolutionizing the ways in which health information is accessed and used by both providers and patients, promising enhancement of quality of care [11] and marking a shift as patients convert from passive recipients to active consumers [7]. eHealth literacy, which is “the use of emerging information and communication technology, especially the Internet, to improve or enable health and health care” [12] (pg 267), is therefore a crucial area of study to understand and enhance the ways in which patients access and use eHealth information.

One measurement tool that is receiving increasing attention in eHealth studies is the eHealth Literacy Scale (eHEALS) [13]. A systematic review of tools to measure eHealth literacy identified 8 different measurement techniques. Noteworthy, however, is that only 1 of these techniques, eHEALS, appears in studies other than the one for which it was designed. Indeed, of 53 published articles, 45 used eHEALS [14]. Clearly, eHEALS is rapidly becoming the accepted standard way to measure eHealth literacy.

However, while there are extensive investigations pertaining to overall health literacy, the eHealth literacy construct and its psychometric properties remain understudied [15-17]. One further review of 19 health literacy instruments, including eHEALS, led to the conclusion that there are insufficient reliability assessments of data collected using health literacy scales. In fact, a key finding of this appraisal was that “limited empirical evidence exists on the reliability and construct validity of health literacy measures. This raises uncertainty about the accuracy of data being produced in relation to health literacy levels at an individual and population level” [18] (pg 367). Unsurprisingly, on this basis came a call for further research.

A further noteworthy omission from current knowledge pertaining to eHEALS is the lack of established measurement invariance. Measurement invariance, which simply means equivalence of measures, is a prerequisite before making any meaningful comparisons between different groups [19]. Indeed, too often researchers assume that an instrument developed for one culture or population automatically measures the same construct across another culture or population. However, without the establishment of measurement invariance, group comparisons are not valid or meaningful [20]. Hence, it is crucial for any scale used extensively across different nations, cultures, and groups to demonstrate measurement invariance. Developed in Canada, the scale has since been used extensively in studies with very different samples and in different cultures, including North America [21], Europe [22], and Asia [23].

Ebbinghaus [24] contended that nation-state formation, international cooperation, and easy availability of data have resulted in some countries being overrepresented (or indeed underrepresented) in many analyses. Consequently, research conducted in 1 country (usually a North American country) is assumed to be relevant to other countries, irrespective of differences in cultural and social forces. This study is part of a larger piece of research into eHealth. The choice of countries emerged from consideration of their very different rankings on health care system performance and their systems of health care provision, in the expectation that patients experiencing these different levels of services, choice, and standards would have different eHealth behaviors. What is important in this study is the basis on which we selected these countries. The Commonwealth Fund [25] ranks countries on the basis of major performance indicators on multiple health care dimensions. The analysis incorporates the views of patients and physicians pertaining to their health care systems, as well as data from the World Health Organization and the Organisation for Economic Co-operation and Development. A private foundation that promotes a good health care system to improve access, quality, and efficiency, the Commonwealth Fund supports independent research on health care issues and focuses primarily on the most vulnerable people in society (low-income people, the uninsured, minorities, young children, and elderly people). The foundation supports independent research on health care issues [25]. The ranking system used by the Commonwealth Fund [25] emerges from analysis of 80 different items pertaining to 6 main dimensions of health care: quality care (effective care, safe care, coordinated care, patient-centered care), access (cost-related problem, timeliness of care), efficiency, equity, healthy lives, and health expenditure per capita.

The United Kingdom was an obvious choice to include in the research because it ranks first overall in the league tables provided by the Commonwealth Fund [25]. While there are still major crisis points with the UK National Health Service [26,27], nevertheless the United Kingdom is ranked first across 8 of the 11 performance areas, including all of the quality-of-care indicators and the efficiency indicator. At the other extreme, ranking bottom overall, is the United States. The United States differs most notably from other industrialized nations in its lack of universal health coverage, but also ranks behind most other countries on key performance indicators pertaining to health outcomes, quality of care, and efficiency of health care delivery. Between these two extremes lies New Zealand, a country where its residents benefit from a public health system that is free or low cost due to heavy government subsidies [28], and where performance rankings are high for health measures such as effective care and coordinated care, but which lags behind many other countries in safety and equity. Notably, New Zealand is a country where eHEALS has never before been used. Hence, the inclusion of such disparate nations in this study is an important contribution to knowledge. Table 1 provides the rankings for each country in the major dimensions and subdimensions of health care provision provided by the Commonwealth Fund [25].

**Table 1:** Commonwealth Fund rankings of health care provision by country.

Dimension or subdimension	New Zealand	United Kingdom	United States
Quality care	4	1	5
Effective care	2	1	3
Safe care	9	1	7
Coordinated care	2	1	6
Patient-centered care	6	1	4
Access	7	1	9
Cost-related problems	6	1	11
Timeliness of care	6	3	5
Efficiency	3	1	11
Equity	10	2	1
Healthy lives	9	10	11
Health expenditure per capita (US $)	3182	3405	8508
Overall ranking	7	1	11

While the 3 countries we selected are vastly different in terms of the Commonwealth Fund health care rankings, they are nevertheless all western countries in which cultures may not differ to the extent that perhaps eastern and western nations may. Nevertheless, comparison between the 3 countries on the major cultural dimensions of national culture [29] reveal that, while they are similar in terms of high indulgence (people in high indulgence societies generally exhibit a willingness to realize their impulses and desires with regard to enjoyment of life, viewing leisure time as important, and spending money as they wish), masculinity (society is driven by competition, achievement, and success rather than caring for others and quality of life), and individualism (self-image is determined by “I” rather than “we,” and personal fulfillment is important), there are some rather large differences. These differences are most notable in terms of long-term orientation, a cultural dimension that measures short-termism and quick solutions over preparing for the future. The latter dimension seems particularly important in terms of health care planning for future generations.

Baby boomers (born between 1946 and 1964) are the focus of this study. Projections suggest that this cohort will place major strains on health care systems in each of these chosen nations [30-32]. Rapid population aging and a steady increase in human longevity are leading to one of the greatest social, economic, and political transformations of all time [33]. Globally, life expectancy has increased by almost 20 years over five decades, and the profundity of this demographic change affects many economic and social areas, including health care. As longevity increases, age-related diseases such as dementia, cardiovascular disease, arthritis, osteoporosis, and type 2 diabetes will place greater demands on health care providers. Hence, in an increasingly technology-driven society, eHealth literacy is a crucially important area of study [34,35]. Many baby boomers are both technologically proficient and increasingly taking a greater role in their own health care [36]. Indeed, baby boomers have a marked difference in social attitudes in comparison with the generation that preceded them, with very different attitudes expressed in certain consumption choices, including bodily maintenance, diet, and exercise [37].

However, statistics show that baby boomers are not particularly healthy. Compared with previous generations, there is a higher prevalence of obesity, alcohol consumption, hypertension, and diabetes among baby boomers in the United States [38]; the vast majority of British boomers have at least one medical condition requiring regular medical care, with only 1 in 6 being condition-free [39]; and few doubt the significant impact that aging is predicted to have on New Zealand’s health care expenditure [40]. Interestingly, the 3 countries under study rank at the bottom in terms of healthy lives (Table 1). One of the performance indicators for healthy lives is healthy life expectancy at age 60 years, and while individual ranking data for this indicator are not provided, it nevertheless gives an insight into the health-related conditions facing the baby boomers under study.

Our study therefore addressed 2 important issues. First, it answered the call for further research to examine the eHEALS, and did this through the use of structural equation modelling to examine its underlying structure. Then, by establishing full measurement invariance, our study validated eHEALS using samples of baby boomers selected from the United States, the United Kingdom, and New Zealand. We begin with a brief overview of the eHEALS and then synthesize the diverse studies that have used the scale. We then argue for the need to establish measurement invariance, before detailing the procedures used to obtain it across these diverse nations. We conclude with a discussion of the implications for future research and practice.

### The eHEALS

Norman and Skinner [41] developed the lily model of eHealth literacy. The lily model depicts 6 core skills or literacies, each represented by an overlapping lily petal that feeds the pistil, which is eHealth literacy. These 6 core skills constitute 2 components. Table 2 outlines this classification of components and provides an overview of each of the core skills.

**Table 2:** Components of eHealth literacy lily model.

Component		Description
**Analytic components: involving skills applicable to a broad range of information sources and contexts**		
	Traditional literacy	Ability to read text, understand written passages, and speak and write a language coherently
	Information literacy	Understand how information is organized on the Internet, how to search for it, and how to use it
	Media literacy	Ability to place information in a social and political context so as to understand how different media forms can shape the conveyed message
**Context-specific components: situation-specific skills**		
	Computer literacy	Ability to use computers to solve problems
	Science literacy	Ability to place health research findings in an appropriate context, thus understanding the research processes involved in knowledge creation
	Health literacy	Ability to read, understand, and act on health information

Shortly after disseminating the lily model of eHealth literacy, Norman and Skinner [13] published the eHEALS, which comprises 8 items designed to “measure consumers’ combined knowledge, comfort, and perceived skills at finding, evaluating, and applying electronic health information to health problems” (pg 1). Norman and Skinner [13] reported sound scale development procedures, describing a process whereby they used the 6 core skills depicted in their lily model to compile an initial pool of items from which “an iterative process of item reduction was used to create an instrument that could be easily deployed within a variety of settings and contexts” (pg 3). This iterative process of item reduction and modification comprised reviews by faculty colleagues, a consumer group with developing literacy skills, and a large pilot test, resulting in the 8-item eHEALS shown in Table 3.

**Table 3:** eHealth Literacy Scale (eHEALS) scale items.

Item number	Description
1	I know what health resources are available on the Internet
2	I know where to find helpful health resources on the Internet
3	I know how to find helpful health resources on the Internet
4	I know how to use the Internet to answer my questions about health
5	I know how to use the health information I find on the Internet to help me
6	I have the skills I need to evaluate the health resources I find on the Internet
7	I can tell high-quality health resources from low-quality health resources on the Internet
8	I feel confident in using information from the Internet to make health decisions

Even from a cursory glance at the scale, it is clear that each item does not relate solely to 1 skills dimension. Rather, though it is not explicit either in the items themselves or in the published scale development article [13], it seems that embedded into each item are several core literacy skills. Item 1, “I know what health resources are available on the Internet,” is perhaps reflecting traditional and computer literacy, while item 7 could incorporate traditional, information, media, science, and health literacies. It is important to note that Norman and Skinner [13] did point out that the eHEALS does not measure the skills directly, but rather is a “measure of consumer’s perceived skills and comfort with eHealth” (pg 5).

Developed and used in further studies in Canada [42,43], the eHEALS has since been used in many countries and cultures across the globe, including the United States [34,44-54], Australia [55], Germany [56], Greece [57], Israel [58], Indonesia [59], Japan [60], the Netherlands [61,62], Norway [63], Portugal [64], Switzerland and Italy [22], Singapore [23], South Korea [65], and Taiwan [66,67], and is being used in an ongoing health intervention study in the United Kingdom [68], although results from this latter study are not yet available. The eHEALS has also been used with a wide variety of samples, including schoolchildren and adolescents [13,45,52,56,64,66,67], parents [48,69], university students [23,42], adults comprising different age groups of a wide age range [16,58,60] and adults comprising solely older generations [34,43,54], as well as veterans [46,70], patients [44,49-51,53,71], caregivers [47], and health service providers [21,59]. The scale has been used with very small (<100) sample sizes [34,42,43,45,59], as well as studies comprising several thousand respondents [48,58,60,66]. Researchers have found eHEALS to be useful for measuring perceptions of eHealth literacy to ascertain skills and training gaps [42] and to measure the success of intervention studies [34,53,68]. The scale has also been beneficial in explaining willingness to adopt personal health record technology [51]. Perhaps even more importantly, though the scale measures self-perceptions of eHealth literacy, higher scores on the scale have indicated good health behaviors, including the likelihood of undergoing cancer screening [60], as well as eating a balanced diet and taking physical exercise [72].

Clearly, eHEALS is becoming an established and well-accepted scale with which to measure eHealth literacy, used across very different studies with a wide range of research questions and a great deal of diversity in terms of sample profiles. However, often the scale is used without due consideration of its validity and reliability. It has been noted that the eHEALS construct does not appear to fully reflect the 6 different types of health literacy [18]; the representativeness of the results from smaller studies has been questioned [73]; and previous authors have noted that the validity of eHealth literacy in general [74], and the eHEALS instrument in particular [62], require further study. Moreover, the original scale authors did note that the eHealth lily model has its roots in social cognitive theory and self-efficacy theory [41]. However, despite their claim that detailed descriptions of these theories appear in their earlier publication [13], there is no explicit mention of these theories or how they were used to develop neither their eHealth literacy definition nor their eHEALS measurement instrument.

### Validity and Reliability of eHEALS

Much of the burgeoning research that has used the eHEALS did so without consideration of the factorial validity of the construct. Of those studies that did examine the measurement properties of the instrument, most used principal components factor analysis [13,50,54,67]. Recently, 1 study examined the construct validity of eHEALS by first using an exploratory components analysis, which extracted 1 factor from 2 different convenience samples. Analysis then turned to further scrutiny of the scale using the Rasch model, which, in addition to providing details about the perceived difficulty of items, provides reliability statistics to estimate how well an instrument separates individuals on the construct. The study concluded that “eHEALS is a reliable and consistent measurement tool for perceived measurement of eHealth literacy. An exploratory factor analysis showed that items loaded on a single factor solution, thereby supporting the criterion of unidimensionality” [75] (pg 11).

However, while exploratory factor analysis such as principal components analysis is very useful for reducing a large number of items to a more manageable amount, a “confirmatory factor analysis of a multiple-indicator measurement model…affords a more rigorous evaluation of unidimensionality according to the constraints imposed by internal and external consistency” [76] (pg 189). Only 2 studies that we know of have used the more complex and sophisticated structural equation modelling to construct a confirmatory factor analysis (CFA) of the eHEALS. The first, conducted in Japan, entailed translation of eHEALS into Japanese [60,77] with CFA used to build a good-fitting model comprising a single factor. The second, a German study [56], compared a single-factor model to a 2-factor model. Of the 2 German alternatives, the 2-factor model was a superior fit, suggesting that the eHEALS is not unidimensional, as claimed in much previous literature, most of which has tended to use principal components analysis. However, as these authors themselves admit, the results of the 2-factor model clearly still did not indicate a well-fitting model because several important indices “indicated a poor model fit” (pg 33). Indeed, even in the better-fitting model, the root mean square error of approximation (RMSEA) was greater than 1.0, which indicates a poor-fitting model [78,79], while the comparative fit index (CFI) of .914 and the Tucker-Lewis index (TLI) of .874 are clearly not close to the .95 needed for a well-fitting model [80].

Noteworthy is that in each of the studies that used CFA, eHEALS was translated into a different language from the English in which it was originally designed. When translated, scale items can take on different meanings, and these nuances can affect perceived meanings for respondents [81,82]. The majority of health information on the Web is not only in English but developed from an English-as-a-first-language cultural perspective, and the ramifications of this appear to be far greater than for English speakers of different ethnic origins [83]. Indeed, in their original presentation of the lily model [41], Norman and Skinner commented on the fact that the overwhelming content of the Web is in English and suggested that English speakers therefore not only are more likely to find eHealth resources that are relevant to their needs, but are also more likely to find eHealth resources that they can understand. Undoubtedly, then, more research needs to examine the unidimensionality of eHEALS in an English-language context.

Importantly, to our knowledge, no previous study has examined the measurement properties of eHEALS in terms of its use with multigroups. To make comparisons between groups, measurement invariance needs to be established. Measurement invariance, or measurement equivalence, is a check to establish that a scale measures the same trait dimension, in the same way, when administered to 2 different groups [84]. Measurement invariance therefore checks that different groups (based on sex, ethnicity, nationality, or any other individual differences) respond to a measurement instrument in similar ways. Too often, researchers make assumptions about measurement equivalence, yet violations of measurement equivalence threaten fundamental interpretations of results [28]. Hence, measurement invariance is essential for testing a theory successfully in different cultural settings [19]. Without such evidence, findings “are at best ambiguous and at worst erroneous” [85] (pg 78). A standard scale, particularly one that exhibits measurement invariance, is a potentially valuable research tool for comparative and longitudinal research purposes in a variety of nations in order to create new theories or test existing hypotheses [86].

There is a growing body of international research that focuses on identifying the antecedents and impact on behavior of the eHEALS. Previous studies have examined the correlates of eHEALS in terms of antecedents such as sociodemographic characteristics [35,44], living arrangements [44], medical conditions and health status [35,44], and frequency of Internet use [44]. Additionally, some studies have attempted to measure behavioral correlates; for example, eHEALS has been described as a marker for consuming more information [87], basic Internet use [62] and using the Internet specifically for health care and lifestyle information [16,23,66], predicting postmedical visit online health information seeking [49], patient willingness to adopt a personal health record [51], and the likelihood of undergoing cancer screening [60]. A growing number of studies are also making comparisons between groups. For example, past research has made direct comparisons of eHEALS scores between different groups on the basis of various sociodemographic variables [44,64,66], and users and nonusers of Web 2.0 for health information [35]. Research has also used eHEALS to identify groups with low and high eHealth literacy and made behavioral comparisons based on these groups [22]. Establishment of measurement invariance of the scale would be a useful contribution to knowledge because measurement invariance is needed to ensure group comparisons are valid and meaningful [20]. Such groups can comprise any distinguishing measure, so to make a comparison of, say, males and females drawn from the same population, measurement invariance of a scale should be checked. This research makes that contribution.

CFA models should test a hypothesis based on a strong theoretical and empirical foundation [88]. As previously discussed, from a theoretical perspective, close scrutiny of the health literacies that make up the lily model (Table 2) and the 8 eHEALS items (Table 3) clearly shows that eHEALS does not reflect the 6 core skills depicted in the lily model. Indeed, this observation appears in previous literatures [56]. Hence, it is not easily apparent how to decide on the number of factors to test in a model based solely on the items in the lily model from which Norman and Skinner [13] claim eHEALS emerged. Norman and Skinner did, however, claim that the “foundations of the eHealth literacy concept are based in part on social cognitive theory and self-efficacy theory which promote competencies and confidence as precursors to behavior change and skill development” [13] (pg 2). It should be noted, however, that although their assertion that these theories are described in detail in their article published that same year [41], this claim does appear to be an overstatement, as there is in fact very little detail pertaining to these theories explicitly in their published work. What these authors did, however, is explain that eHEALS is based on the premise that the core skills or literacies in the lily model (Table 2) are not static and can be improved with intervention and training. In fact, they explained that literacy is as much a process as it is an outcome. It is here that social cognitive theory is apparent in their work, as social cognitive theory is based on a model of causation where behavior, environmental influences, and personal factors (which include cognitive, affective, and biological factors) all interact and influence each other [89]. Hence, rather than the lily model, here we used the underlying theories to eHEALS, namely social cognitive theory and self-efficacy theory [41], to attempt to develop a hypothesis upon which a measurement model can be tested.

The root of social cognitive theory is the concept of reciprocal determinism, where 3 factors—person, environment, and behavior—are interlinked [89]. The individual learns from experiences and the environment, which incorporates external social contexts. Responses to this learning and the environment affect the individual’s behavior and therefore their ability to achieve goals. As Bandura [89] stressed, diversity in psychobiological origins, experiential conditions, and behavior results in substantial individual differences in what individuals can and cannot do. This theory therefore makes perfect sense as a foundation to eHEALS, given that individuals differ greatly in their competences pertaining to the literacies depicted in the lily model.

It is clear that eHEALS measures an individual’s perceived skills as opposed to actual skills. An important influence in the personal dimension of the reciprocal model of social cognitive theory is self-efficacy, as this can directly influence self- motivation. Self-efficacy relates to self-belief and confidence; hence, self-efficacy is not to do with the skills a person has, but rather what that person believes they can achieve with those skills. Self-doubt and negativity can lead to failure, while self-belief and confidence can lead to an increase in effort and persistence until success is realized. Hence, self-efficacy can lead to restructuring of goals, including either lowering standards or setting higher goals to achieve even greater things, all based on the individual’s perceived capabilities [90].

Attempting to apply these theories to the eHEALS is not straightforward at first glance. Nevertheless, it is relatively easy to identify those items that relate to self-efficacy. Items 6 (“I have the skills I need to evaluate…”), 7 (“I can tell high-quality….from low-quality…”), and 8 (“I feel confident in using…”) all appear to pertain to a belief and confidence in one’s own evaluation skills to effectively use health resources and information. However, keeping in mind that some previous empirical evidence suggests that eHEALS is neither a single-factor structure nor a 2-factor structure [56], the remaining items require close scrutiny to identify potential groupings. This close scrutiny reveals a difference between items 1 and 2, which both pertain to an awareness of what resources and information are available on the Internet, and items 3-5, which all pertain to the “how” in terms of how to find and how to use these resources. In other words, items 1 and 2 relate to an awareness of Internet health resources, items 3-5 related to the skills needed to access them, and items 6-8 relate to the self-belief that one can effectively evaluate them.

**Figure 1:**
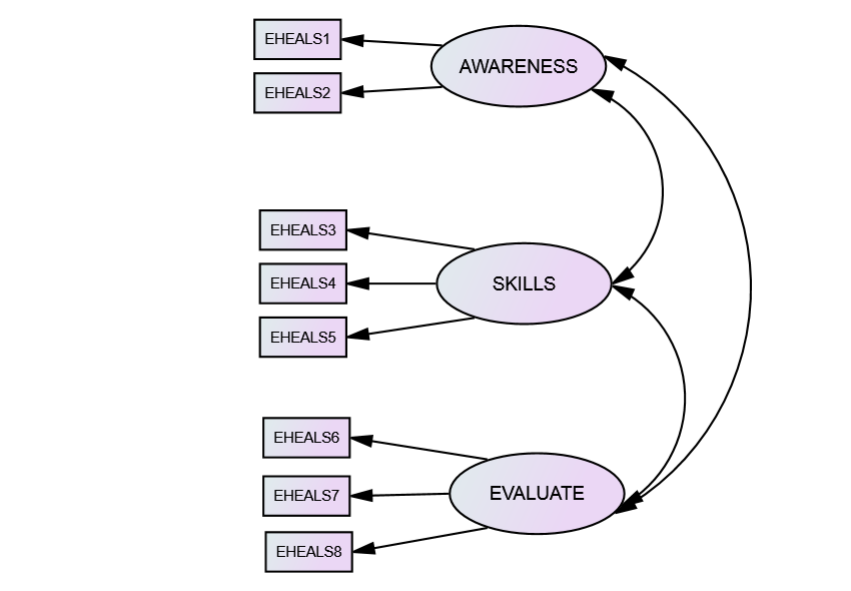
eHealth Literacy Scale (EHEALS) 3-factor model. Factor 1 pertains to awareness of what resources are available and where they are (items 1 and 2 in the scale), factor 2 pertains to the skills and behavior needed to access them (items 3-5), and factor 3 pertains to believing one has the ability to evaluate them once accessed (items 6-8).

These 3 groupings do, in fact, relate to social cognitive theory in that social and technological changes affect life experiences to different degrees among different individuals [89]. Hence, knowledge of such social and technological innovations (various levels of awareness and learning about health resources on the Internet), which are reflected in items 1 and 2 of the scale, are clearly influenced by environmental factors that affect exposure to different sources of information pertaining to Internet health resources. Then, the skills needed to access these Internet health resources, which comprise items 3-5, are affected by modelling, instruction, and social persuasion in the environment. Clearly there is a behavioral element here, and such skills are a response to environmental stimuli, as well as being affected by personal factors such as internal dispositions, motivation, and biological properties that impose constraints on capabilities. This reciprocity is a key aspect of social cognitive theory [91]. Finally, self-efficacy is clearly apparent in the remaining items (items 6-8), as these items reflect an individual’s self-perception of the skills needed to fully utilize the eHealth information attained on the Internet. Of course, the individual’s environment and previously learned knowledge will influence the levels of self-belief that the individual holds, which is in line with the reciprocal nature of social cognitive theory. Figure 1 shows the resulting 3-factor model to be tested. Factor 1 pertains to awareness (knowledge of what resources are available and where they are), factor 2 pertains to the skills and behavior needed to access them, and factor 3 pertains to believing one has the ability to evaluate them once accessed.

## Methods

### Instrument

The original eHEALS was developed at a time before the rise of social media [9]. Extensive social networking opportunities, as well as advances in technology such as Web 2.0, change the landscape in terms of how consumers interact with health information [92]. Hence, we tweaked the wording of the original scale items to incorporate “health information” as well as “health resources.” This is because we felt that solely using the term “resources” may limit eHealth information search to official resource sites (eg, the American Cancer Society, Cancer Research UK, or Cancer Society NZ) and not incorporate the increasingly important electronic word-of-mouth that occurs on social media sites and online forums. Norman [9] advocated that the scale may need to be adapted, suggesting a social media subscale could perhaps enhance the current scale, while others have suggested that interactive applications would indeed enhance the eHEALS [93]. For these reasons, we added the words “and information” to several items. Informal feedback from friends, family, and colleagues when we asked them to name some “Internet health resources and information” reflected a wide perspective, in that people immediately cited search engines (usually Google) but also cited a wide variety of other sources, including online forums and Facebook support groups. For example, one person who was at the time undergoing tests for multiple sclerosis replied that he had not only studied the Multiple Sclerosis Society of Great Britain’s webpages and viewed this as an important health resource, but also joined a Facebook group to learn more about how people coped with their diagnosis, and he viewed this as an informal information resource. Hence, rather than drastically changing the scale by adding items specific to social media, we hoped that information gained from social media would now be incorporated. Table 4 shows the adapted scale.

Approval to conduct the study was granted by the ethics committees of the University of Liverpool, the University of Waikato, and the University of Lugano.

**Table 4:** Adapted eHealth Literacy Scale (eHEALS).

Item number	Description
1	I know what health resources and information are available on the Internet
2	I know where to find helpful health resources and information on the Internet
3	I know how to find helpful health resources and information on the Internet
4	I know how to use the Internet to answer my questions about health
5	I know how to use the health information I find on the Internet to help me
6	I have the skills I need to evaluate the health resources and information I find on the Internet
7	I can tell high-quality health resources and information from low-quality health resources and information on the Internet
8	I feel confident in using information from the Internet to make health decisions

In addition to the tweaked eHEALS, because the study is part of a larger piece of research into eHealth, the survey contained questions pertaining to information search and usage such as sources of health information used (including interpersonal sources such as friends and family, as well as formal health information sources such as nonprofit organizations and health care providers), perceived advantages of using Internet eHealth sources (eg, 24-hour accessibility, convenience, anonymity), and perceived usefulness of Internet eHealth resources in comparison with information provided by health care providers (with a Likert-type scale ranging from “much less useful” to “much more useful”). In addition, the questionnaire contained a battery of sociodemographic variables, including age (measured via year of birth), sex (male or female), marital or relationship status (married; widowed; divorced; separated; in a domestic partnership or civil union; single, but cohabiting with a significant other; and single, never married), work status (employed full-time, employed part-time, retired, unemployed, homemaker, on government or state benefit, student or in training, other: please specify), and educational attainment (university degree; vocational training, eg, trade apprenticeship, professional qualification, college qualification; high school; less than high school).

### Sample

In each country, we commissioned a commercial organization to survey randomly selected baby boomers. A prerequisite for completing the survey was that respondents (1) had to be born between 1946 and 1964 and (2) had used the Internet to search for health information in the last 6 months. Each organization was instructed to collect data from at least 250 baby boomers, and therefore the first respondents were included in the survey before the survey was closed; hence, the surveys were open for less than 2 days in each country. Prior to completing the survey, respondents were informed of its purpose (an international research project studying the use of the Internet to search for and share health information), its academic nature, how the data would be stored (password-protected secure university drives) and for how long, and the length of the survey, which typically took 20 minutes to complete. This procedure resulted in 996 usable questionnaires. There were no missing data, as a “not applicable” option was given to suitable questions, and while respondents were able to review and change their answers, they were unable to submit incomplete questionnaires.

### Data Analysis

To further check the psychometric properties of the eHEALS, we conducted a series of CFAs using IBM SPSS Amos 20 (IBM Corporation). We used standard global model fit indices with well-known fit guidelines. Hence, we used the RMSEA, which is a popular measure of fit in structural equation modeling and is now recognized as one of the most informative criteria in structural equation modeling [94]. We adhered to the guidelines suggested by Hu and Bentler [80]; therefore, RMSEA values of .00 to .05 indicate a close or good fit, .05 to .08 a fair fit, .08 to .10 a mediocre fit, and over .10 a poor fit. Other fit indices that we used to assess the models were the CFI and TLI, both of which should be close to .95 [80]. The Akaike information criterion (AIC) is a fit statistic used to compare 2 models, with smaller values indicating better fit [95].

Additionally, we used probability of close fit (PCLOSE) to test the hypothesis that RMSEA is good in the population, testing the null hypothesis that RMSEA is no greater than .05 [94]. In other words, PCLOSE is an additional test of model fit, and this result indicates a close fit. Data analysis also included the use of Hoelter critical N, which is another fit statistic that differs from the others used here in that it focuses directly on the adequacy of the sample size, rather than the fit of the model. A “value in excess of 200 is indicative of a model that adequately represents the sample data” [94] (pg 83).

Steenkamp and Baumgartner [85] contended that multigroup CFA is the most powerful and versatile approach to testing for cross-national invariance and offered a sequential testing procedure for doing so. We followed this procedure here. Measurement invariance comprises 3 levels: configural, metric, and scalar. Each level is an increasingly stringent test of multigroup invariance. Consequently, we constructed a multigroup measurement model and tested it first for configural invariance, which provided a baseline model for comparisons of subsequent tests for invariance. Testing the pattern of salient (nonzero) and nonsalient (zero or near zero) loadings defined the structure of the measurement instrument [85]. In other words, the purpose of the test of configural invariance was to explore the basic structure of the construct and check that participants from different groups conceptualized the constructs in the same way [20]. Simply put, did respondents, irrespective of their cultural or national heritage, employ the same conceptual framework [96] when answering the questions that make up the eHEALS?

Configural invariance does not, however, mean that the respondents in different nations reacted to the scale items in the same way. To compare item scores meaningfully across nations, and thus have confidence in observed item differences being indicative of cross-national differences in the underlying construct, metric invariance is required. Indeed, for a scale to be useful in larger studies that examine structural relationships with other constructs cross-nationally, metric invariance is needed. [85]. Metric invariance checks that the scale is measured in the same way across groups, in that not only do different groups respond to scale items in the same way, but also the strength of the relations between items and their underlying construct is the same across groups [20].

In practice, most researchers focus on the 2 preceding and most fundamental steps, which are tests of configural and metric invariance [97]. There may be some projects, however, where researchers want to compare means and, to do this, the scale needs to exhibit scalar invariance. Scalar invariance implies that cross-national differences in the means of the observed items are due to differences in the means of the underlying constructs [85,98], and therefore indicates that the latent means can be meaningfully compared across groups [20]. Scalar invariance tests whether, in addition to the factor loadings, the intercepts are also the same, which implies that cross-national differences in the means of the observed items are due to differences in the means of the underlying constructs [98].

## Results

### Preliminary Analysis

Table 5 provides a profile of the sample by country.

**Table 5:** Sample profile by country (N=996).

Characteristics		United Kingdom (n=407)	New Zealand (n=276)	United States (n=313)	Total
**Sex, n (%)**					
	Male	192 (47.2)	141 (51.1)	163 (52.1)	496 (49.8)
	Female	215 (52.8)	135 (48.9)	150 (47.9)	500 (50.2)
Age in years, mean (SD)		59.6 (5.15)	61.3 (5.78)	60.3 (5.35)	60.3 (5.43)
**Work status, n (%)**					
	Working full-time	132 (32.4)	82 (29.7)	84 (26.8)	298 (29.9)
	Working part-time	63 (15.5)	54 (19.6)	32 (10.2)	149 (15.0)
	Retired	130 (31.9)	67 (24.3)	114 (36.4)	311 (31.2)
	Unemployed/welfare	35 (8.6)	42 (15.2)	27 (8.6)	104 (10.4)
	Homemaker	35 (8.6)	12 (4.3)	20 (6.4)	67 (6.7)
	Other	12 (2.9)	19 (6.9)	36 (11.5)	67 (6.7)
**Educational attainment, n (%)**					
	Less than high school	3 (0.7)	0 (0.0)	8 (2.6)	12 (1.2)
	High school	158 (38.8)	89 (32.2)	59 (18.8)	305 (30.6)
	College/practical/technical/occupational	148 (36.4)	101 (36.6)	101 (32.3)	350 (35.1)
	University degree	98 (24.1)	86 (31.2)	145 (46.3)	329 (33.0)

Table 6 provides the mean eHEALS item scores by country. While the purpose of this study was not to compare the countries in question in terms of eHealth literacy (that will be done elsewhere), noteworthy is that even a cursory glance at Table 6 reveals that US respondents had higher scores than their New Zealand and UK counterparts. We do not know whether this was due to the overall higher educational attainment of the US sample (Table 5), perceptions of poorer health care provision (Table 1), or other reasons. Across all 3 countries, the corrected item-total correlations revealed no low values (all were >.635) and it was not possible to obtain a higher alpha score by deleting any item. In all 3 nations, Cronbach alpha results were very high (.931 for the United Kingdom, .917 for the United States, and .910 for New Zealand). Indeed, with medical researchers being urged to be more critical when reporting alpha values [99], alphas this high (>.90) may suggest redundancies or that the construct being measured is too specific [100]. Hence, our analysis turned to further investigation using CFA.

**Table 6:** Mean eHealth Literacy Scale (eHEALS) item scores by country.

Item	United States		United Kingdom		New Zealand	
	Mean	SD	Mean	SD	Mean	SD
1	3.81	0.76	3.67	0.77	3.56	0.81
2	3.91	0.71	3.78	0.71	3.70	0.77
3	4.01	0.68	3.80	0.71	3.88	0.66
4	3.96	0.77	3.83	0.72	3.81	0.68
5	3.89	0.73	3.71	0.74	3.73	0.70
6	3.62	0.94	3.47	0.82	3.37	0.93
7	3.61	0.85	3.48	0.87	3.28	0.93
8	3.66	0.79	3.50	0.88	3.39	0.94
Cronbach alpha	.917		.931		.910	

### Confirmatory Factor Analysis

The first step in testing for discriminant validity of a model structure with multiple latent factors is to reject the possibility of a single-factor structure [101]. Table 7 details these single-factor CFA results.

**Table 7:** eHealth Literacy Scale (eHEALS) confirmatory factor analysis by country: single-factor structure.

Country	n	χ^2^	*df*	*P* value	RMSEA^a^	PCLOSE^b^	AIC^c^	CFI^d^	TLI^e^
United Kingdom	407	379.003	20	<.001	.210	<.001	411.003	.864	.809
New Zealand	276	263.140	20	<.001	.210	<.001	295.140	.833	.767
United States	313	199.218	20	<.001	.169	<.001	231.218	.896	.854

^a^RMSEA: root mean square error of approximation.

^b^PCLOSE: probability of close fit.

^c^AIC: Akaike information criterion.

^d^CFI: comparative fit index.

^e^TLI: Tucker-Lewis index.

The data did not fit the 1-dimensional model well. In addition to significant chi-square values (χ^2^_20_=379.003, *P*<.001 for the United Kingdom; χ^2^_20_=263.140, *P*<.001 for New Zealand; and χ^2^_20_=199.218, *P*<.001 for the United States), the RMSEA values of .210 for the United Kingdom and New Zealand and .169 for the United States fell outside the guidelines [78,79] proposing that values less than .05 indicate a good fit, values ranging from .05 to .08 reflect a reasonable fit, values between .08 and .10 indicate a mediocre fit, and values greater than .10 reflect a poor fit. Likewise, the CFI and TLI should be close to .95 [80], yet fell well below the cutoff point suggested for these indices in all 3 nations.

Our analysis then turned to examination of the hypothesized 3-factor model, using the UK data. Testing for factorial equivalence encompasses a series of hierarchical steps that begins with the determination of a baseline model for each group separately [94]. The first step, then, was to establish a baseline model from 1 of the samples. We chose the UK data simply because the UK sample comprises the largest number of respondents. While the 3-factor model revealed a much better fit to the 1-dimensional model, examination of the modification indices suggested improvement through the pairing of error terms associated with eHEALS items 2 and 3. One possible method effect that can trigger error covariance is a high degree of overlap in item content [94]. The high Cronbach alpha scores presented in Table 6 do of course suggest such redundancy [100]. Scrutiny of items 2 and 3 did reveal a degree of overlap, in that item 2 asks respondents if they know where to find resources, while item 3 asks them if they know how to find these resources. Clearly, to some people, there is not much difference in the meaning of these questions. Given the apparent overlap in the content of these items, and the high Cronbach alphas, which had already suggested some redundancy between scale items, we respecified the 3-factor model to include these correlated errors, and analysis moved from confirmatory to exploratory mode.

The RMSEA of .066 was within the range for a reasonable-fitting model, the CFI of .989 and the TLI of .981 far exceeded the recommended minimum values of .95, and the AIC of 84.174 shows a dramatic improvement on the previous model. Examination of the standardized residuals revealed none to exceed the threshold of 2.58 [102]; indeed, the highest standardized residual was 1.102 between eHEALS5 and eHEALS8, with all other standardized residuals falling below 1. In sum, the respecified 3-factor model fitted the UK data well.

### Measurement Invariance

For the scale to be useful in multinational research, measurement equivalence is needed; without evidence of invariance, conclusions based on the scale “are at best ambiguous and at worst erroneous” [85] (pg 78). The next goal, then, was to examine the basic meaning and structure of the construct cross-nationally, to establish whether the scale is conceptualized in the same way across countries. Before moving to analysis of multinational invariance, however, Byrne [94] recommended testing the model separately in each group as the first step toward multigroup CFA. Table 8 gives the goodness-of-fit indices for each nation (including the UK data for comparative purposes). All samples demonstrated indices falling within the boundaries outlined above. Therefore, the model fit was acceptable for all countries.

**Table 8:** eHealth Literacy Scale (eHEALS) confirmatory factor analysis by country: 3-factor structure.

Country	n	χ^2^	*df*	*P* value	RMSEA^a^	AIC^b^	CFI^c^	TLI^d^
United Kingdom	407	44.174	16	<.001	.066	84.174	.989	.981
New Zealand	276	40.651	16	.001	.075	80.651	.983	.970
United States	313	43.529	16	<.001	.075	83.529	.984	.971

^a^RMSEA: root mean square error of approximation.

^b^AIC: Akaike information criterion.

^c^CFI: comparative fit index.

^d^TLI: Tucker-Lewis index.

We then constructed a multigroup measurement model (based on the final 3-factor model) and tested it first for configural invariance. Table 9 shows the results of this and subsequent analyses. The fit indices of the configural model (χ^2^_48_=128.363, *P*<.001, RMSEA=.041, CFI=.986) indicate that the model cannot be rejected, which led to the conclusion that the specification of the items that index the 3 factors of eHEALS are configurally invariant for the 3 nations under study.

**Table 9:** Measurement invariance of the eHealth Literacy Scale (eHEALS) across New Zealand, the United States, and the United Kingdom.

Model	χ^2^	*df*	*P* value	RMSEA^a^	PCLOSE^b^	Δχ^2^	Δ *df*	Significance	CFI^c^	ΔCFI	Critical N	
											.05	.01
1) Configural invariance	128.363	48	<.001	.041	.954	N/A^d^	N/A	N/A	.986	N/A	505	571
2) Metric invariance	149.262	58	<.001	.040	.983	20.899	10	.022	.984	.002	512	573
3) Scalar invariance	203.237	74	<.001	.042	.971	74.874	26	<.001	.978	.008	466	515

^a^RMSEA: root mean square error of approximation.

^b^PCLOSE: probability of close fit.

^c^CFI: comparative fit index.

^d^N/A: not applicable.

Table 9 also presents the results of the metric invariance analysis, when all factor loadings are constrained equally across all 3 groups. Despite the fact that metric invariance is often difficult to achieve [97], although the chi-square change between the configural and the metric model is nonsignificant, the ∆CFI of .002 is well below the proposed cutoff point of .01 [103], suggesting that the measurement model is completely invariant. This means result provides strong evidence that the eHEALS is ready to use, with a degree of confidence, in the different countries under study.

Indeed, the scale is now ready for exploring and testing structural relationships, which is the most important application for most researchers. Despite the fact that full invariance is often difficult to achieve [97], as Table 9 shows, further analyses demonstrated the eHEALS to exhibit scalar invariance; hence, analysis can include direct comparisons of mean scores. Indeed, both the “excessively stringent” [94] (pg 220) test of invariance resulting in a significant value in the change in chi-square (74.874, ∆df=26, *P*<.001), and the ∆CFI of .008 was below the .01 cutoff point [103]. Hence, despite potential social or cultural differences, the scale is unaffected. For each model, the RMSEA closeness of fit (ie, PCLOSE) far exceeds the minimum recommended *P* value of .05 [104], and Hoelter critical N at both the .05 and .01 values are greater than 200.

Despite not checking for normality prior to analysis, it is noteworthy that the data indicated no departure from normality, as evidenced by no rescaled β2 values exceeding 7 [105]. Table 10 provides these rescaled β2 values. However, there was some suggestion of multivariate kurtosis. Consequently, bootstrapping using 2000 bootstrap samples, none of which was unused, revealed only very small differences between the maximum likelihood-based estimates and the bootstrap-based estimates (Table 10). Moreover, no confidence intervals included zero (Table 11). Thus, there were no substantial discrepancies between the results of the bootstrap analysis and the original analysis, and the interpretations of the results presented above are without fear that departure from multivariate normality has biased the calculation of parameters [106].

**Table 10:** Rescaled β2 values and differences in maximum likelihood estimates and bootstrap estimates in the revised eHealth Literacy Scale (eHEALS) for New Zealand, the United States, and the United Kingdom.

eHEALS item	Rescaled β2 values			Differences in maximum likelihood estimates and bootstrap estimates		
	UK	NZ	US	UK	NZ	US
1	0.741	0.238	1.415			
2	2.011	1.304	1.850	.008	0.013	0.007
3	2.110	1.997	2.924			
4	1.986	1.296	2.181	0.002	0.008	0.006
5	1.709	0.746	0.934	0.003	0.008	0.015
6	0.189	0.016	0.297			
7	0.136	–0.117	–0.063	0.005	0.001	0.012
8	0.484	0.078	–0.085	0.005	0.002	0.013

**Table 11:** Bias-corrected bootstrap confidence intervals for the revised eHealth Literacy Scale (eHEALS).

Parameter	United Kingdom	New Zealand	United States
eHEALS1 ← awareness	0.878-1.055	0.923-1.203	0.921-1.129
eHEALS2 ← awareness	1.000-1.000	1.000-1.000	1.000-1.000
eHEALS3 ← skills	0.926-1.083	0.792-1.007	0.783-0.989
eHEALS4 ← skills	0.928-1.083	0.856-1.069	0.975-1.129
eHEALS5 ← skills	1.000-1.000	1.000-1.000	1.000-1.000
eHEALS6 ← evaluate	0.862-0.995	0.874-1.098	1.093-1.448
eHEALS7 ← evaluate	0.829-0.972	0.912-1.133	0.973-1.283
eHEALS8 ← evaluate	1.000-1.000	1.000-1.000	1.000-1.000

Finally, we tested convergent validity. First, inspection of the factor loadings presented in Table 12 revealed that all exceed the ideal of .7 [107]. Moreover, all factor loadings were positive and statistically significant.

**Table 12:** Standardized regression weights^a^for the revised eHealth Literacy Scale (eHEALS).

Parameter	United Kingdom	New Zealand	United States
eHEALS2 ← awareness	.919	.846	.912
eHEALS1 ← awareness	.836	.825	.857
eHEALS5 ← skills	.843	.841	.842
eHEALS4 ← skills	.877	.857	.867
eHEALS3 ← skills	.874	.832	.877
eHEALS8 ← evaluate	.843	.837	.818
eHEALS7 ← evaluate	.795	.832	.751
eHEALS6 ← evaluate	.854	.826	.730

^a^All factor loadings are positive and statistically significant.

Additionally, Table 13 presents the average variance extracted (AVE) and the construct reliability (CR) results for each country. All AVEs exceeded the cutoff of .5 [108], indicating convergent validity, and all CRs exceeded .7, indicating good reliability. Taken together, the evidence provides support for the convergent validity of the 3-construct eHEALS measurement model.

**Table 13:** Average variances extracted (AVE) and construct reliability (CR).

Parameter	United Kingdom		New Zealand		United States	
	AVE	CR	AVE	CR	AVE	CR
Awareness	.772	.871	.699	.822	.783	.878
Skills	.748	.898	.711	.881	.743	.897
Evaluate	.691	.870	.691	.871	.589	.811

**Figure 2:**
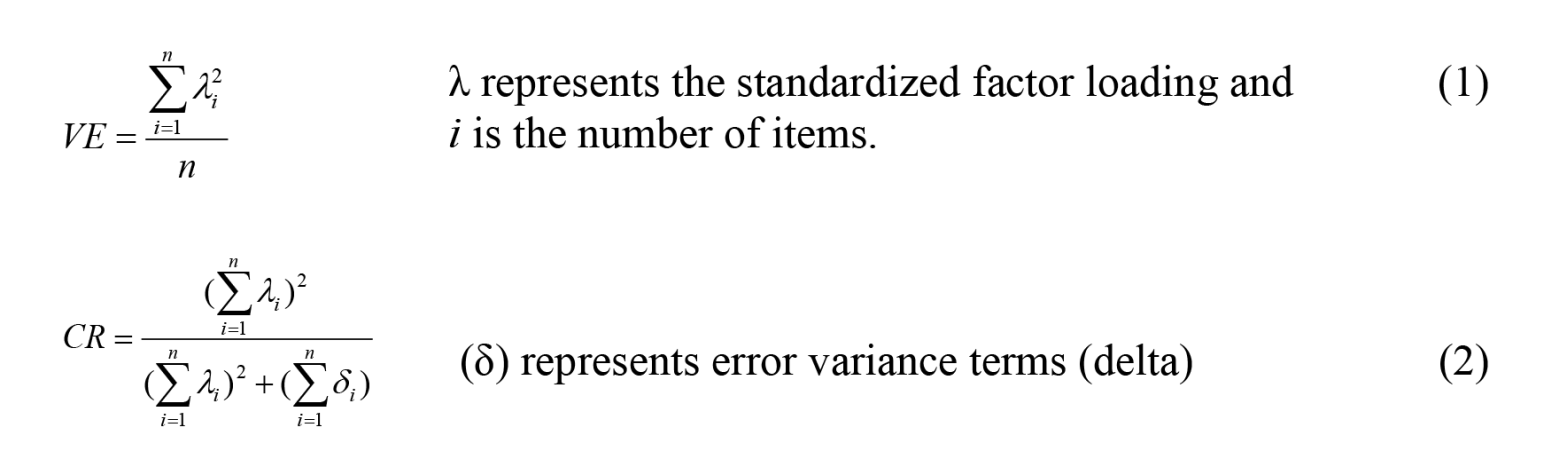
Formulae for calculating (1) average variance extracted (VE) and (2) construct reliability (CF).

The AVE and CR are not provided by IBM SPSS Amos software, so we calculated them using the formulae shown in Figure 2.

## Discussion

### Principal Findings

The finding that eHEALS comprises 3 distinct factors is a novel and important one. The eHEALS measure was developed based on the lily model of eHealth literacy, which had earlier been advanced based on social cognitive theory and self-efficacy theory [13,41]. However, to our knowledge, no previous research has ever fully examined these theories to analyze the measurement properties of the eHEALS. Despite this omission, a burgeoning body of research uses eHEALS as a measure of the extremely important concept that is eHealth literacy. As more and more of the world’s population gains Internet access, and as patients increasingly expect to be active rather than passive consumers of health care services [2], the concept of eHealth will continue to grow in importance. Health care providers and researchers need a valid, reliable, and easy-to-use measurement tool with which to assess levels of perceived eHealth literacy among different groups of patients. Until now, there has been some debate about the construct validity of the eHEALS, and indeed the validity of the measurement of eHealth in general [15-18], casting doubt over subsequent results. Hopefully this study alleviates some of that doubt.

The 3 factors that emerged here are clearly based on the underlying theory on which Norman and Skinner’s [41] definition of eHealth literacy is founded. From the perspective of social cognitive theory, behavioral capability refers to knowledge and skills needed to influence behavior. Additionally, human competence needs self-belief in the ability to use those skills effectively [91]. The 3-factor model presented here clearly reflects these aspects of the theory. The first factor comprises items relating to knowledge about health resources and information that are available on the Internet. The second factor relates to the skills needed to access and use the health resources and information. Finally, the third factor relates to levels of self-belief in the ability to use this information effectively.

The 3-factor structure presented here is the first to demonstrate that eHEALS does indeed relate to the social cognitive theory upon which it is founded. Future research should attempt to do the same. Indeed, all too often insufficient tests of dimensionality, reliability, and validity mar many past research studies, and it is hoped that researchers using the eHEALS measure will in future give due consideration to these crucially important dimensions of any measurement instrument. Previously, research has not given due attention to the underlying theoretical arguments for unidimensionality versus multidimensionality.

It is, of course, possible that different results have emerged here due to different populations from those that have informed past research. While eHEALS is used extensively in the United States [34,44-54], no previous study, to our knowledge, has ever used eHEALS in New Zealand, and the results of the 1 known study where it is being used in the United Kingdom [68] are not yet published. The majority of previous research that has examined the factor structure of eHEALS has used principal components analysis [13,23,50,54,64,67], rather than using CFA, which provides a much more rigorous evaluation than does principal components analysis.

Interestingly, the 2 studies that have used CFA have examined the scale after it was translated into languages other than English, the one in which it was designed. The study that found a single factor using CFA had translated eHEALS into Japanese [60,77], while the other, which found 2 factors, had translated the scale into German [56]. Hence, our study is the first to examine the factorial validity of eHEALS in the English language, in which it was originally designed. Given that translation problems can arise [81,82], it is possible that language issues have affected results in other studies. Moreover, in this study the minor tweaks to the scale in terms of insertion of the words “and information” into 5 of the items could have affected respondents’ derived meaning. Finally, the use of samples comprising solely baby boomers could have affected results. Of the 2 previous studies to use CFA, the first used a wide age range [60,77] and the second used adolescents [56]. Of course, eHEALS was originally designed using 13- to 21-year-olds [13]. We recommended that future research take these issues into account.

No previous studies that we know of have attempted multinational measurement invariance of eHEALS. Establishment of full measurement invariance is therefore another novel and important contribution. The results of a configural invariance test suggest that the respondents under study employ the same conceptual framework when answering eHEALS, despite their different cultural experiences and indeed very different experiences of health care provision.

In addition, despite it being often difficult to achieve [97], metric invariance was established. This result suggests that eHEALS is measured in the same way across these nations. Steenkamp and Baumgartner [85] (pg 82) noted that “When the purpose of the study is to relate the focal construct to other constructs in a nomological net, full or partial metric invariance has to be satisfied.” Clearly, the level of measurement invariance required for the purposes of investigating eHealth literacy in a variety of disparate nations is established.

Finally, studies may be needed to examine cross-national comparisons of the eHEALS mean scores. For such comparisons to be valid, scaler invariance needs to be established. Certainly, a cursory glance at the mean scores presented in Table 6 suggests that US baby boomers are more eHealth literate than their UK and New Zealand counterparts. While the examination and discussion of such differences is beyond the scope of this paper, it is nevertheless important to note that such comparisons can now be made legitimately, and confidence in the results of such comparisons has increased due to the establishment of full measurement equivalence.

Dolnicar and Grün [109] noted that results are only as good as the data on which they are based. Given the extensive use of eHEALS in research to date, the lack of prior research to establish measurement invariance is surprising. The results presented here therefore make an important contribution to knowledge, as “without evidence of measurement invariance, the conclusions of a study must be weak” [110] (pg 119). In establishing full measurement invariance, comprising configural, metric, and scalar invariance, this study has demonstrated that eHEALS is now ready to use with confidence in these diverse nations. Moreover, the AVE and the CR results for each nation all suggest convergent validity and good reliability. Overall, these results provide solid support for the convergent validity of the 3-factor eHEALS model.

In addition to the implications of this study for theory, these findings are important from a practical perspective. Results demonstrate that, consistent with the theory on which it was developed, eHEALS assesses self-perceptions of 3 important and distinct (though interrelated) elements of eHealth literacy: awareness of Internet health resources (items 1 and 2), the skills needed to access them (items 3-5), and the self-belief that one can effectively evaluate them (items 6-8). Hence, eHEALS can now be used to segment health consumers into distinct groups based on their scores on the scale, with corresponding intervention and training provision designed around meeting the needs of these segments. Those individuals with relatively low scores on the awareness factor would need to be offered basic training designed to address the rudimentary elements of eHealth in terms of describing and demonstrating the range of appropriate resources available and how they can be found. For people whose scores are relatively low on this factor, such training should perhaps be stand-alone and could be the foundational level of training. Once they master these skills, individuals could be offered the second level of training, designed for those people whose scores are relatively low on the skills factor. This skills training should be designed to perhaps build on basic knowledge and concentrate on developing the individual’s search and evaluation skills pertaining to eHealth resources. Finally, a third training program could be developed that concentrates on developing and building self-efficacy, to give people the self-belief that they are truly empowered patients who are able to play an active role in their own health care. Most training and educational programs incorporate levels of progression in their design, and eHealth intervention and training programs should be no different.

Practical intervention and training around eHealth is important for several reasons. eHealth has the potential to assist self-management in people with chronic health conditions, and evidence suggests that even in developed countries, half of the population with chronic health conditions have elementary navigational needs and would benefit from basic training in this area [55]. Training programs are crucial because patients with higher levels of health literacy have significantly lower anxiety levels than people with inadequate health literacy, and have fewer and shorter consultations with health care providers [111]; hence, there are economic benefits to such training programs. Improvements in ability and self-belief to access and use Internet health resources have cumulative benefits in terms of ability and willingness to use other eHealth resources such as electronic health records, patient portals, and self-management tools [74]. Thus, understanding different skill levels and needs is important for policy makers and health care providers, who could all use such information to develop correct and targeted interventions for different segments of the population. Indeed, it has even been suggested that eHealth is so important that it should be incorporated into school curricula [57]. When they first designed eHEALS in 2006, Norman and Skinner [13] claimed that the scale has the potential to identify those who may or may not benefit from referrals to an eHealth intervention or resource. Our research builds on this claim and suggests that eHEALS can be used to ascertain the type of intervention or resource that could benefit these different segments.

### Limitations

The study is not without its limitations. First, while baby boomers are a justifiably important sample for health care and eHealth research, the 3-factor structure that emerged here needs to be investigated using younger samples to ensure that boomers are not unique and the 3-factor structure is indeed applicable to all age groups. Second, while the 3 nations we chose do vary a great deal in terms of health care provision rankings and to a lesser extent on some important cultural dimensions, they are nevertheless all English-speaking western countries. It has been noted that when eHEALS was translated, different factorial structures emerged. We recommend that the 3-factor model be tested in very diverse cultures (eg, eastern countries) and among non-English-speaking nations. Third, we acknowledge that the original version of eHEALS was designed before the rise in social media and Web 2.0 technology. While we made some attempt to incorporate the interactive nature of today’s online environment by tweaking the scale (specifically, adding “and information” to items), the suggestion that the marginally updated version used here is sufficient to incorporate interactive resources is based solely on anecdotal evidence gained by asking family, friends, and colleagues. We recommend that a more formal study investigate the way respondents perceive the eHEALS in its revised form, as it may need to be more extensively altered, or indeed a new scale may need to be designed, in order to fully capture the myriad of interactive eHealth resources that health care consumers are now able to access.

### Conclusions

The usefulness of a short, easy-to-administer scale that measures a person’s perception of their eHealth literacy is beyond doubt. Indeed, the extensive use of the eHEALS across a variety of studies in countries across the globe is testimony to the urgent requirement for such an instrument. The research presented here details a more rigorous investigation of the measurement properties of the eHEALS than has previously been conducted, using CFA rather than principal components analysis. Based on social cognitive theory and self-efficacy theory, a 3-factor model was tested and confirmed.

Research often needs to make comparisons across groups or across time and, to be able to do this, a scale must demonstrate measurement invariance. Only by establishing measurement invariance can there be assurance that comparisons are valid [112]. In other words, establishing measurement invariance provides evidence that score differences across countries are a true representation of differences in the construct under study, rather than differences brought about by social and cultural factors or other such confounding variables [96]. This research has demonstrated full measurement invariance of the eHEALS among baby boomers in 3 diverse nations, meaning the scale is now ready to use with far more confidence by researchers in these nations. This research has therefore added weight to Norman and Skinner’s [13] contention that the scale is a useful addition to a range of eHealth assessments, from primary care to health promotions. The identification of 3 distinct factors not only confirms the theoretical antecedents on which eHEALS was built, but also suggests that the scale can now be used to better segment health care consumers and identify different skills gaps, enabling policy makers and health care providers to design and offer tailored interventions and training programs to address such gaps.

Over 80% of baby boomers in all 3 countries under study use the Internet regularly [113-115]. Nevertheless, this cohort did not grow up using the Internet, and there may be some for whom knowledge, skills, and self-confidence around eHealth resources still lag behind the levels that perhaps exist among younger cohorts. Yet the baby boomer cohort is crucially important from an eHealth perspective because forecasts predict that this cohort is increasingly going to put major pressures on health care systems [30-32]. Importantly, Bandura [91] explains that personal factors can be altered dramatically to improve the functioning of individuals. Competency can be developed through training and guidance, which in turn can increase self-belief in capability levels. While eHealth training lessons are already available across all 3 countries we studied, the findings suggest these training programs should be built around knowledge of what health information and resources are available on the Internet, and then developing the skills needed to access them. Motivational enhancements should also be incorporated into such training to ensure an enhancement in self-belief.

In sum, this study fills an important gap in that it provides future researchers and practitioners with more faith in the eHEALS than existed previously. The scale can now be used with a degree of confidence in a variety of nations and in studies with a variety of research objectives, including the modeling of complex relationships among variables. The choices of nations and the demographic of the samples therein are also strengths of the study: all too often, scale evaluation and development comprises young (often student and often US) samples. Studies often use scales developed in a different country or culture without checking that the measure is equivalent. This study has demonstrated that eHEALS can be used with confidence across a variety of nations and cultures. This study therefore lends support for the contention that eHEALS is a valid scale with which to measure self-perceptions of eHealth literacy, a concept that is set to become even more important in the future.

## Abbreviations

AIC: Akaike information criterion
AVE: average variance extracted
CFA: confirmatory factor analysis
CFI: comparative fit index
CR: construct reliability
eHEALS: eHealth Literacy Scale
PCLOSE: probability of close fit
RMSEA: root mean square error of approximation
TLI: Tucker-Lewis index
